# Volunteers in a biography project with palliative care patients – a feasibility study

**DOI:** 10.1186/s12904-019-0463-0

**Published:** 2019-10-07

**Authors:** Michaela Hesse, Simon Forstmeier, Henning Cuhls, Lukas Radbruch

**Affiliations:** 1 Department of Palliative Medicine, University Hospital Bonn, Sigmund-Freud-Str. 25, D- 53127 Bonn, Germany; 2 Department of Psychology, University Siegen, Adolf-Reichwein-Str. 2a, D- 57076 Siegen, Germany; 3 Department of Palliative Medicine, Malteser Hospital Seliger Gerhard Bonn / Rhein-Sieg, Von-Hompesch-Str. 1, D- 53123 Bonn, Germany

**Keywords:** Palliative care, Volunteers, Biography work, Psychosocial interventions

## Abstract

**Background:**

Increasing the quality of life with short interventions for vulnerable patients is one of the objectives of palliative care. Biographical approaches are used in a range of different interventions which may require considerable resources of staff time and energy. This study evaluated the feasibility of training hospice volunteers in biographical interviews of patients confronted with a life-limiting disease. For the purpose of this study, we evaluated resources such as time needed for training, coordination and supervision, outcome such as completion of the intervention in appropriate time and risks such as causing distress in patients or volunteers as major determinants of feasibility.

**Methods:**

Nine volunteers from a hospice service attended an advanced training with an introduction to palliative care, biography work, interview techniques, transcribing and writing. Volunteers interviewed a patient and developed a written narrative from the interview. Volunteers completed a questionnaire before training and were interviewed at the end of the project. The interviews were audiotaped, transcribed, and evaluated using descriptive and qualitative content analysis.

**Results:**

Patients provided positive feedback from the intervention. Volunteers felt that their involvement was personally rewarding and were moved by the courage and confidence of the patients. There were no systematic problems or negative experiences reported neither by volunteers nor by patients.

**Conclusions:**

We found the use of volunteers for biography work with patients in palliative care feasible and effective in this study. Volunteers needed supervision and ongoing support in providing this intervention.

## Background

Diagnosis of a life-limiting disease is a disruptive experience. In consequence patients often feel that life is divided in the time before diagnosis and the time with the illness. Patients need to cope with their illness and need to realign their identity accordingly. Palliative care is a holistic approach, with all health care professionals in the team in close communication with the patient and his caregivers looking for resources supporting the patient [1]. Narratives are an important tool to construct meaning, and there is a growing body of literature and research in the use of narratives in palliative care [2–5]. Telling stories is an intrinsic part of human beings. An alleviating effect may be achieved by a nonjudgmental and interested listener [2]. Volunteers` engagement with patients is a key resource [6] and is characterized by informality [7] for they stand for unintentional awareness. Volunteers in palliative care prove responsibility of society and are crucial to guarantee future growth of hospice and palliative care as stated in the review of Candy et al. [8] Involvement in biographical interventions meets the categories `being with´ and `doing for´ facilitating well-being as described by Dodd et al. [9] The roles of volunteers can be described as ambiguous and this should not imply quasi-professional roles or substitutive roles [7]. Ambiguity should be a plus and stand for flexibility and patient-orientation.

Different concepts using biographical elements have been proposed such as Short-Term Life Review [10], Life Review [11, 12], Life Completion Therapy [13], Meaning-Making Intervention [14], Living with Hope Program [15], or Dignity Therapy [16, 17]. Biographical interventions have been shown to increase the quality of life and alleviate depression [13, 18–22]. However, psychosocial interventions such as biography work are not widely implemented. Sustainability of such interventions is challenging due to high staff costs [17, 23]. Fitchett et al. [17] describe Dignity Therapy as costly and time-consuming and discuss the question who should provide the intervention. Keall et al. [23] elicit the limited implementation from perception of time and resources. They see potential in administering interventions by trained volunteers.

In some countries such as Australia, New Zeeland, United Kingdom biography interventions are not provided by health care professionals, but by hospice biographers or volunteer biographers [1, 24, 25]. Volunteers are an indispensable feature in hospice and palliative care. They are well established in most countries [26]. A randomized controlled study from Allen et al. [27] demonstrated that senior volunteers were able to deliver a reminiscence and creative activity intervention in an outpatient setting. Looking at the experiences of the volunteers with a qualitative approach [28] revealed a positive impact and personal benefit for the volunteers. The authors see a need for further research in the realm of implementation and in the long-term impact on volunteers.

In Germany, it is estimated that more than 40.000 volunteers contribute to hospice and palliative care. Volunteering has a longstanding tradition in Germany as a major part of hospice care. Training for hospice volunteers in Germany comprises a total of 100 to 120 h focused predominantly on reflection on motivation, but also on qualification and work experience. Volunteer tasks are described in the training curriculum as developing a trusting relationship with the patient, accompanying the patient and the family, providing psychosocial support, dealing with death and dying, facilitating communication and helping with social, ethical, and spiritual issues [29].

Volunteers could provide a huge manpower resource for psychosocial interventions such as biography work. However, adequate training and supervision has to be ensured. The intervention used in this study was a short open biographical approach to foster story-telling. Patients could choose the topics they want to talk about. This intervention is in the style of Romanoff and Thompson [2] who claim that narrations about life or illness have potential to seek for meaning.

The overall aim of this study is to examine if a biographical intervention provided by skilled hospice volunteers is feasible regarding the following domains: i) resources: do we have the resources for training, coordination and supervision; ii) outcome: can the volunteers finish the intervention comprising the interview and the writing in an appropriate time, and iii) risks: are there indications for distress in patients or volunteers.

## Methods

The Methods` section is organized along the TIDieR Checklist [30].

### Aim

The overall aim of this study is to clarify if implementation of a volunteer provided intervention with a biographic approach is possible. We tested a short open biographical approach as “unstructured autobiographic storytelling “ [2, 31]. We were looking for a sustainable concept to implement this intervention.

### Design

The study was designed as an open uncontrolled mixed methods study, evaluating qualitative and quantitative data in a small convenience sample.

### Setting

The intervention took place on the palliative ward of University Hospital Bonn. The ward has a cooperation agreement with hospice service `Bonn Lighthouse´.

### Participants

Volunteers were recruited by the coordinator on a supervision group meeting. Inclusion criterion was training as a hospice volunteer was completed (120 h plus supervision of first cases). Patients were recruited on the Palliative ward of University Hospital Bonn by one of the authors (MH). Inclusion criteria were: > 18 years, German speaking, no psychiatric diagnosis, no mental constraints.

### Intervention

We tested an “unstructured autobiographic storytelling “(Webster, Romanoff) delivered by volunteers. Studies have demonstrated that biographical interventions improve quality of life and decrease depression in palliative care patients [10, 13, 20–23, 32–34]. Implementation in clinical practice is sparse. Volunteers have been able to deliver a reminiscence intervention [27]. However, the open question is if implementation of a volunteer-based intervention is feasible.

### Materials

[1] Baseline evaluation with closed questions was used before the training in biographical work for volunteers. The structured questionnaire included seven questions about their skills, four questions on their appraisal of biography work, and six questions about their motivation (see Additional file 1) [2]. At the end of the project we performed a focus group interview. All volunteers were asked to talk about their experience along open-ended questions on the following issues: preparation, the interview itself, transcribing procedure, writing and their evaluation. (see Additional file 2)

### Procedures

Written consent and the self-administered questionnaire [1] were collected from each volunteer before starting the training. Training, intervention and evaluation were performed in three phases.

### Phase 1 (training)

Volunteers were trained in biography work for 14 h. This consisted of an introduction unit (2 h) and three workshops lasting four hours. Learning goals included knowledge about influence of illness, goals of palliative care, importance of storytelling as a coping method, and options for biography work. Technical skills included active listening, mirroring answers and reflecting emotions, and skills to handle audiotapes and transcription software. Self-experience took a major time share so that volunteers were reflective to their own biography and learned how to handle challenging interview situations. Volunteers were taught to come to patients as a writer not as an investigator. Supervision was provided during the whole time and volunteers were accompanied in every phase of the project.

### Phase 2 (intervention)

All volunteers were informed when a patient had given informed consent to participate in the project. If volunteers could meet the patient in the following week they confirmed by mail. At the day of the interview they had a short briefing and received a dictaphone. One of the authors (MH) accompanied the volunteer to the patient, introduced them to each other, and left the room putting a `Do not disturb´ on the door. After the interview the volunteer came to MH and returned the dictaphone. The interview file was copied on an USB-stick and given to the volunteer. The volunteer received a short debriefing. The volunteer was asked to take notes of the experience and document the time needed. MH then visited the patient to get his feedback on the intervention.

### Phase 3 (evaluation)

At the end of the project the participating volunteers were invited to a focus group interview. This session contained two rounds. In the first round each volunteer talked about his/her actions along the questions [2]. In a second round all volunteers were invited to discuss and exchange their experiences. The focus group interview was audiotaped and transcribed verbatim.

### Who provided

Skilled Hospice volunteers from a hospice volunteer service `Bonn Lighthouse´.

**How** Every volunteer interviewed a patient face to face and constructed a written narrative from the audiotaped interview which was then presented to the patient as a little booklet or a letter. The initial question asked was: “What do you want to tell? What shall I write down for you?”

**Where** Patients were recruited on the Palliative ward of University Hospital Bonn.

**When and How much** Data were collected between January and July 2018. Preparation and training took place from January till March 2018. Biography interventions were performed from April till June 2018. Interventions were planned in one or two sessions depending on the health status of the patient. However, all patients wanted to tell their story in one session. The focus group was held on the 25th of July 2018.

Supervision was provided during the whole time and volunteers were accompanied in every phase of the project. Adherence to the training content was checked by supervisor. The palliative care team includes a psychologist and volunteers as well as patients were made aware of this resource in case of distress related to the intervention. The study was approved by the ethical committee of University Hospital Bonn (no. 368/17).

**Qualitative Data Analysis** was performed by two of the authors (MH, HC) using the software MAX-QDA 11. Data were analysed using the descriptive and qualitative content analysis [35]. The main themes of the evaluation were inductively applied, and data were sorted into categories. Quality criteria of qualitative research are replicability, triangulation and reliability [36, 37]. The data was coded independently by two researchers for reasons of intercoder reliability [37]. For reasons of replicability interpretation of data was structured thoroughly and categories were defined and discussed till consensus was reached. For triangulation, categories from the analysis were compared with the items of the questionnaire.

## Results

Nine volunteers participated in the project and each interviewed one patient. Eleven patients were asked to participate in the feasibility study. Two patients dropped out due to health decline (Table 1).

**Table 1:** Sample

Voliunteers		Patients	
Gender	2 men, 7 women	Gender	5 men, 4 women
Age	Mean 46 (range 23–65)	Age	Mean 62 years (range 30–83)
Volunteering in hospice	Mean 4.6 years (range 1–13)	Disease	
		Cancer	*n* = 7
		Non-cancer	*n* = 2 (CHF = 1, genetic disorder = 1)
Martial status		Martial status	
married/living with a partner	*n* = 5	married/living with a partner	*n* = 4
divorced	*n* = 1	divorced	*n* = 3
single	*n* = 3	single	*n* = 1
		widowed	*n* = 1
Highest education level achieved		Highest education level achieved	
University degree	*n* = 6	University degree	*n* = 2
A-level	*n* = 2	A-level	*n* = 1
Secondary-school	*n* = 1	Secondary-school	*n* = 5
		Elementary school	*n* = 1

### Resources

Organisation of the interviews was time consuming, requiring coordination resources for matching patients and volunteers, finding time slots for the interviews, providing informed consent and completing questionnaires. Supervision required briefing and debriefing of the volunteers. All in all, organisation and supervision of nine biography interviews took 28 work hours. The whole project required 9 months.

### Outcome

All volunteers managed to interview a patient and write a story or letter in appropriate time so that all patients got their written document. Seven interviews took place in the palliative care unit and two at the patients´ home due to discharge from hospital. Interviews lasted between one and two hours, on average 80 min. Transcriptions required between 2.75 and 8 h, with an average of 5.65 h. Volunteers wrote the stories in two to 4 days. Stories were handed back to the patients on average 11 days after the interview (range 2 to 28 days) with 28 days being a discordant value due to holidays of the patient.

Volunteers responded in the questionnaire that their motivation was to improve communication skills (*n* = 8), to have a new scope of activity (*n* = 8) and for altruistic reasons (*n* = 6). Five volunteers said they received additional qualification with the training and improved their technical skills. All volunteers were interested in other people’s life story. Four volunteers felt very well and five well trained in communication. Seven said they were good listeners and two rather good listeners. Five volunteers answered that they come easily into contact with other people and four said rather easy. Four volunteers expected difficulties in the interviews, described as “to come to an end”, “flow of conversation”, “handle emotions” and to “help the patient to cope with intense feelings”. None of the volunteers expected difficulties in writing up the story. Prior to the workshops eight volunteers appraised that patients would benefit from biographical interviews and five said that they would use this offer themselves.

Preparation of the interview was only described by two volunteers, one who wrote down some questions, the second had some exercise with the technical equipment. The others reported no preparation.

*“I made some notes, what kind of questions could I ask, what initial introduction could I give.” (VLG2) “Prepared- just in terms of technical device.” (VAZ6).*

The interview itself was described as ´no-brainer´. *“I have not said a lot.” (DND1) “She talked a lot”. (DLG2) “He had great need to talk”. (DHZ3) “I have not asked anything. He talked to me more than an hour.” (DAZ5)* One volunteer pointed out*“We laughed a lot. It was even funny.” (DGH10).*

Assessment of transcription and writing was non-homogenous. Five volunteers reported no problems:

*“It took a long time, but it was a great experience. The patient came so near with every word.”(TND1) “I could just type word for word and it was him.” (THZ5) “I did not have to change anything.” (TAZ7) “I typed it completely and at the most, besides two or three small bits, I did not change anything.” (TGV8).*

Four volunteers reported difficulties, two struggled with technical problems due to hardware. The others complained about the time and the compilation of the story.

*“I had difficulties to download the program on my Mac.” (TKH4) “It was very time consuming. I needed another program because of Apple.” (TGH9) “There were a lot of different aspects and to make a text was difficult for me.” (TLG3) “It was heavy going- one word at a time.” (TE6).*

In the interview volunteers shared self-criticism as well as a description of their experience. Self-criticism was related to attentive listening and volunteers felt that it would have been better to ask less and to keep silence.

*“Already in the moment I asked the question, I felt it was dispensable.” (SFE2) “I am thankful for the moments where I managed to keep silent. It were long breaks, but he needed the time. I really had to learn that.” (SND5) “I have not guided him in the interview except once, when I asked to tell about the relationship.” (SVG6) “When I listened to the audiotape I thought it would have been better to have talked even less. `Hmm´ and `yes´ would have been sufficient.” (SND7).*

Experience and perception of the patient was described, especially the closeness in the shortness of time.

*“It was an intense experience.” (END1) “It was amazing, very, yes very. He became very close in the interview.” (EAZ2) “I felt it was a gift.” (EFE4) “The patient was very close emotionally. It was touching.” (END5) “I got more and more deep respect for the patient … she opened up her life for me.” (ELG8) “I was impressed and fascinated and really affected, that his main concern in his illness was his family.” (EHZ11) “The moment and the interview itself is an intimate moment, this is very precious.” (EVG18) “She was filled with thankfulness looking back. She said she enjoyed the interview and appreciated it. She appeared composed and placid at the end, this way I would describe it.” (EGH20).*

### Risks

All patients gave positive feedback and were very satisfied with the intervention. Patients could choose what they wanted to tell and what they wanted to ignore. Therefore we did not evoke bitter memories. The majority of volunteers in this pilot study were very experienced. Accompanying patients and having trustful conversations is more related to attitude rather than technical skills. However, biography interviews require a different attitude compared to other conversations volunteers are having with patients and caregivers, for example the need to listen rather than talk. The advanced training in biography work deepened communication skills and self-experience in that regard. In supervision and evaluation volunteers assessed the training to be sufficient and felt well prepared for the biography interviews.

One volunteer needed a consultation with the supervisor after the interview to cope with the patients´ fate. The other volunteers used supervision to discuss the interview looking for improvements in their interactions. We found no indication for causing distress.

## Discussion

### Resources

The main goal of this study was to test the feasibility of a biography intervention delivered by trained volunteers. Within addition to the resources needed for the biography training, coordination and supervision required three work hours per interview, which was in the order we had estimated.

### Outcome

Patients appreciated the interest and the attention they received in the project. The results of the randomized controlled study of Allen et al. [27] confirm that volunteers could be trained to deliver an intervention achieving improvements in outcome similar to the results found with other providers. High motivation and dedication of the volunteers were perceptible throughout the whole project. Volunteers were very reflective and demonstrated capability for introspection. However, comparing the answers in the questionnaire with the guided interview some discrepancies in self-reported skills and appraisal of biography work were seen. Even though volunteers characterized themselves as good or rather good listeners in the questionnaire the evaluation of the interviews highlighted that listening was not attentive enough and that silence was hard to bear. In contrast, four volunteers expected difficulties in the interview in the questionnaire, but the evaluation of the interviews showed no such problems. None of the volunteers expected difficulties in writing the story but four volunteers reported technical difficulties, time exposure, and problems with the outline of the story.

### Risks

Patients chose the topics they wanted to talk about in the biography interviews. The selection of themes might have been influenced by the setting with an interviewer being a volunteer. We discussed if traumatic events might be relieved by recalling the past. However, patients provided positive feedback. None of the volunteers reported problems in the interview two patients even dictated a letter. Similarly, most interviews did not cause emotional distress in the volunteers, and only one intervention was needed from the supervisor. Our findings correspond with the findings of Allen et al. [27] who stressed even positive health benefits to the volunteer.

### Volunteers

Chochinov et al. used a biography intervention as a core component of the dignity therapy for palliative care patients [16]. However, they restricted the provision of dignity therapy to trained health care professionals such as counsellors, psychologists, social workers, chaplains, doctors, nurses, and care workers [38]. The authors maintained that volunteers are not capable of performing biographical interviews. In contrast, volunteer biographers have been reported in New Zealand. Training volunteer biographers comprised application and self-assessment and half-day training. The training consisted of experiential learning, exercises and discussion. Biographers had monthly supervision group meetings. The benefit of the intervention which is established for 17 years was described as development of compassionate, respectful relationships upholding the dignity and well-being of the patients.

Palliative care claims to provide holistic care, which requires consideration of body, mind and spirit of the patients, for all these dimensions contribute to a sense of wholeness [39]. However, there is an ongoing discussion which professionals should cover these dimensions in the multi-professional palliative care team. For the biography intervention a comparison with the delivery of spiritual care – which can be administered by generalists such as nurses or physicians or specialists such as chaplains - might be helpful regarding the question of provider. In the biopsychosocial-spiritual model of care physicians should not restrict themselves to prescription of medicines, but also consider spiritual care [40], in the collaborative model each member of the team has to provide spiritual care as part of interdisciplinary care [41]. In the study from Hanson et al. the effectiveness of spiritual care did not differ by professional groups. They found a range of different professionals such as chaplains, nurses, physicians, social workers, family, or friends providing spiritual care and reported a holistic perception of spiritual care independent of the profession of the provider [42]. Extrapolating these results to biography work, volunteers might be well suited to address the patients´ needs to reflect on their life. However, this means that volunteers may have to be an integral part of the multidisciplinary team in order to provide effective biography work.

Unique characteristics of volunteering are having time, freedom of being without specific remits and the ability to spend their whole-hearted attention. The adequate availability of time was found to be crucial in administering interventions by Keall et al. who looked at feasibility of life completion interventions by nurses. They even concluded that training in counseling was beneficial but not essential [43]. In our study patients felt esteemed and might have found meaning and worth as discussed by Lichter et al. [25] Training volunteers in biography work not only provided a valuable addition in the scope of interventions, but also offered an interesting and enriching new task for volunteers. Burbeck et al. described ambiguity, flexibility and informality as main characteristics of volunteering [7]. They pointed out that volunteers are an integral part and an economic column in palliative care and described quasi-professional roles. Therapeutic interventions such as music therapy or art therapy usually are not part of the regular health care package. In Germany, sickness funds will not reimburse costs for creative arts interventions, so that these interventions require additional funding. We are planning to continue with the biographical intervention using trained volunteers from the volunteer service. This will not be done as a clinical trial, but data from the ongoing quality management documentation can be evaluated descriptively with a larger sample size in the future.

The engagement of trained volunteers can ensure sustainability of the provision of biography work – as well as any other psychosocial intervention. As Allen et al. [27] pointed out reflect involving volunteers a high monetary value and they demonstrated the ability of volunteers delivering a reminiscence intervention. However, volunteers cannot substitute professionals and have no specific therapeutic remit. One volunteer needed debriefing to cope, and coordination was needed to set up patients and volunteers for the interview, highlighting the need for professional supervision and coordination to minimize risks for patients and volunteers.

Further research is needed to assess the burden on patients and volunteers as well as the effectiveness of volunteer-led biographical interventions in studies with larger numbers of participants in order to evaluate the cost-effectiveness of the intervention.

### Limitations

The sample of volunteers was small and homogeneous regarding education and skills, and selection bias for the volunteers was high. In consequence, the results are not representative for palliative care patients nor for hospice volunteers. Further limitation of the study is due to translation. However, we did not aim for representativeness, as the main purpose was to demonstrate feasibility of biography training and intervention. With only one volunteer and no patient reporting distress following the interview risk was low. Larger samples may be required to evaluate the impact of the additional burden with time needed for transcription of the interviews and supervision on volunteers` motivation, as well as for evaluation of effectiveness and risks.

## Conclusions

This study confirmed that biography training for volunteers was feasible and enabled volunteers to perform biographical interviews with palliative care patients. The engagement of trained volunteers can ensure sustainability of the provision of biography work. Patients appreciated the attention they received, and volunteers felt that their involvement was personally rewarding. Volunteers required supervision and ongoing support in providing this intervention.

## Supplementary information

**Additional file 1.** Material 1 Questionnaire.

**Additional file 2.** Material 2 Appraisal.

## Data Availability

The transcripts of the interviews are stored at the Department of Palliative Medicine of the University Hospital Bonn. However, due to data protection regulations these data cannot be shared.

## Abbreviations

MAXQDA: Software for qualitative and mixed methods research
