# Supplementary material for: Volunteers in a biography project with palliative care patients – a feasibility study
Source: BMC Palliat Care. 2019 Oct 7;18:79. doi: 10.1186/s12904-019-0463-0 (PMC6781359; doi:10.1186/s12904-019-0463-0)
Supplement: Supplementary file 1 — Additional file 1. Material 1 Questionnaire. [file 12904_2019_463_MOESM1_ESM.docx]

## Questionnaire for Volunteers

**1. Demographic Information**

| **Gender** | **❑ female** | **❑ male** |
| --- | --- | --- |
| **Age** |  | |
| **Profession** | **❑ self-employed**  **❑ employed** | **❑ retired** |
| **Marital status** | **❑ single**  **❑ married**  **❑ living with a partner** | **❑ divorced**  **❑ widowed** |
| **Highest education level achieved** | **❑ Secondary school - GCSE** | **❑ Further education - A-level**  **❑ University** |
| **Volunteering in hospice since:** |  | |

**2. Interests/skills**

|  | **Disagree** | **Partially disagree** | **Partially agree** | **Agree** |
| --- | --- | --- | --- | --- |
| **2a I am interested in other people’s life stories** | ❑ | ❑ | ❑ | ❑ |
| **2b I am confident in managing conversations** | ❑ | ❑ | ❑ | ❑ |
| **2c I do not want to know personal and private things about patients** | ❑ | ❑ | ❑ | ❑ |
| **2d I am a good listener** | ❑ | ❑ | ❑ | ❑ |
| **2e I easily make contact with others** | ❑ | ❑ | ❑ | ❑ |
| **2f I learn from the experiences of others** | ❑ | ❑ | ❑ | ❑ |
| **2g I have a good feeling for language** | ❑ | ❑ | ❑ | ❑ |

**3. Expectations from biography work**

|  | **yes** | **no** | **Not sure** |
| --- | --- | --- | --- |
| **3a I think it is good for the patients** | ❑ | ❑ | ❑ |
| **3b I expect difficulties during the interview** | ❑ | ❑ | ❑ |
| **If yes, which** |  | | |
| **3c I expect difficulties in writing the patients’ stories** | ❑ | ❑ | ❑ |
| **If yes, which** |  | | |
| **3d I would make use of the biography offer myself** | ❑ | ❑ | ❑ |

**4. Motivation**

|  | **yes** | **no** | **irrelevant** |
| --- | --- | --- | --- |
| **4a I’ll be getting another qualification** | ❑ | ❑ | ❑ |
| **4b I will improve my communication skills** | ❑ | ❑ | ❑ |
| **4c I will improve my technical skills** | ❑ | ❑ | ❑ |
| **4d I will expand my scope** | ❑ | ❑ | ❑ |
| **4e I’m doing it for altruistic reasons** | ❑ | ❑ | ❑ |
| **4f Other:** |  | | |
