# Supplementary material for: Volunteers in a biography project with palliative care patients – a feasibility study
Source: BMC Palliat Care. 2019 Oct 7;18:79. doi: 10.1186/s12904-019-0463-0 (PMC6781359; doi:10.1186/s12904-019-0463-0)
Supplement: Supplementary file 2 — Additional file 2. Material 2 Appraisal. [file 12904_2019_463_MOESM2_ESM.docx]

## Interview appraisal

## Interview preparation:

- - Did you prepare for the interview? If yes, how?
  - Where do you see possible advantages/disadvantages in preparing for the interview?
  - Did you have any problems during preparation?
  - Do you see any need for improvement?

Conducting the interview:

- - Describe your thoughts and feelings during the course of the interview (How did you feel?)
  - What impressions concerning the state of your partner did you get during the interview? How did your partner appear to be to you?
  - What was easy /difficult in conducting the interview?
  - Did you have any problems during the interview?
  - Do you see any need for improvement?

Follow-up and writing:

- - How did you organise the follow-up and the writing of the story?
  - What was easy/ difficult?
  - Did you have any problems?
  - Were you satisfied with the result? What challenges did you face?
  - Do you see any need for improvement?

Feedback:

- - Do you think the intervention was good for the patient? If yes, in what manner?
  - Did you feel well prepared?
  - Would you have liked anything in addition? Was anything missing?
  - Could you imagine working as a volunteer biographer?
  - …………..
